# PRDX5 as a novel binding partner in Nrf2-mediated NSCLC progression under oxidative stress

**DOI:** 10.18632/aging.102605

**Published:** 2020-01-03

**Authors:** Xinming Chen, Xiang Cao, Weizhang Xiao, Ben Li, Qun Xue

**Affiliations:** 1 Department of Thoracic Surgery, Affiliated Hospital of Nantong University, Nantong 226001, Jiangsu, China

**Keywords:** peroxiredoxin 5, nuclear factor-related factor 2, NSCLC, oxidative stress

## Abstract

Non-small-cell lung cancer (NSCLC) is one of the most common malignant tumors in the world. Reactive oxidative species (ROS) and nuclear factor-related factor 2 (Nrf2) -antioxidant response element (ARE) signal pathway are known to play important roles in the development of NSCLC. In this study, we identified Peroxiredoxin 5 (PRDX5) as a novel binding partner for Nrf2. PRDX5 was significantly increased in human NSCLC specimens and cell lines. Nrf2 interacted with PRDX5 in H_2_O_2_-stimulated NCSLC cells, and the interaction promoted the expression of NAD(P)H: quinone oxidoreductase 1 (NQO1) protein in NSCLC cells. Further, high expression of Nrf2 and PRDX5 were associated with worsened prognosis in patients with NSCLC significantly. Moreover, animal studies showed that the growth of tumors treated with Nrf2 and PRDX5 shRNA was significantly lower than that of the other groups. All these data indicated that overexpressed PRDX5 in NSCLC promoted binding with Nrf2 and enhanced NQO1 expression and NSCLC development. Overall, our studies demonstrated that PRDX5 can be a novel binding partner of Nrf2 in promoting NCSLC development under oxidative stress and provide potential opportunity for improving NSCLC therapy.

## INTRODUCTION

Lung cancer is a kind of common malignant tumor, and its mortality rate ranks first in the world [1]. In all types of lung cancer, non-small cell lung cancer (NSCLC) accounts for more than 80% of the cases. The initial understanding of the development mechanism of lung cancer is mainly based on the exploration of smoking and/or other harmful substances that inducing cancer. But recent studies have shown that even in the United States, where smoking rate significantly dropped, the incidence of lung cancer is not changed obviously [1, 2]. Although the prognosis of patients with lung cancer has been improved in recent years, the survival time is still very short, and the 5-year survival rate is less than 15% [3]. Therefore, it is of great significance to explore the new pathogenesis and therapeutic strategy of NSCLC.

Based on the mechanism of tumorigenesis, development, treatment and prognosis, in addition to the traditional proto-oncogene, the role of reactive oxygen species (ROS) is a hot research field in tumor biology. ROS includes H_2_O, O_2_^-^, OH^-^ and so on. It is produced by mitochondrial electron transport chain including cytochrome P450, lipoxygenase and NADPH oxidase [4, 5], and plays its normal and pathophysiological functions in cells [6]. Besides oxidative damage to proteins and nucleic acids, ROS can also act as a second messenger to regulate gene transcriptions [7]. In tumor cells including lung cancer cells, ROS levels are higher than that in normal cells, mainly because the abnormal metabolic level in tumor that leads to excessive production of ROS in hypoxic-ischemic environment. While in tumor therapy, many anticancer drugs are also achieved by changing the intracellular ROS level [8, 9]. Therefore, it is an important target in researching of tumor pathogenesis and treatment.

Nuclear factor-related factor 2 (Nrf2) is an important cellular protein which gets activated in response to oxidative stress and protects normal cells against oxidative damage. Paradoxically, upregulation of Nrf2 has been found to provide growth advantage to many cancer cells by promoting cell proliferation and providing protection against oxidative stress and anticancer agents, thus contributing to chemoresistance [10, 11]. Constitutive activation of Nrf2 due to loss of function mutation in the Nrf2 inhibitor Keap-1 leads to increased chemoresistance in NSCLC [12]. However, the molecular mechanism underlying Nrf2-mediated NSCLC progression remains to be clarified.

To further elucidate the role of Nrf2 in NSCLC tumorigenesis, we identified PRDX5 (Peroxiredoxin 5) as a novel binding partner for Nrf2 through immunoprecipitation in tissues and lung cancer cells. PRDX proteins belong to the superfamily of antioxidant proteins and are widely distributed in prokaryotes and eukaryotes. The PRDX family of lactating animals consists of 6 members: PRDX1-PRDX6. PRDX5 was first reported in 1992, mainly located in cytoplasm, mitochondria, peroxisome and nucleus [13]. Some studies have shown that PRDX proteins can reduce peroxide and superoxide through thioredoxin, can be induced by ROS/JNK pathway under oxidative stress, and has a strong antioxidant and scavenging effect on free radicals. [14, 15]. For the functional study of PRDX5, the researchers also found that its expression is related to cell proliferation, differentiation and signal transduction and so on [16–18]. Moreover, PRDX-1,3,4 and 5 were found to be expressed in more than 80% of breast cancer cases, and the expression of PRDX5 was related to tumor size and lymph node metastasis [19]. However, there were few reports about PRDX5 in lung cancer.

NAD(P)H: quinone oxidoreductase 1 (NQO1) is a very important enzyme that regulates intracellular redox status and binds to its receptor NADPH cause oxidative stress and redox cycles, resulting in the degradation of toxic substances by active steroids and their derivatives. Some studies have shown that Nrf2/NQO1 signaling pathway plays an important role in oxidative stress in respiration, digestion, nerve, cardiovascular system and other organs, and the expression level and activity of NQO1 are closely related to the occurrence and development of tumor [20–22].

In the present study, we found that PRDX5 was significantly increased in human NSCLC specimens and cell lines. We showed that Nrf2 interacted with PRDX5 in H_2_O_2_-stimulated NCSLC cells, and the interaction promoted the expression of NQO1 protein in NSCLC cells. Further, the effect of Nrf2/PRDX5 interaction on cell proliferation was evaluated using NSCLC cell cultures. Moreover, high expression of Nrf2 and PRDX5 were associated with worsened prognosis in patients with NSCLC significantly. All these data indicated that overexpressed PRDX5 in NSCLC promoted binding with Nrf2 and enhanced NQO1 expression and NSCLC progression under oxidative stress.

## RESULTS

### PRDX5 interacts with Nrf2 in NSCLC tissues and cell lines

**Figure 1:**
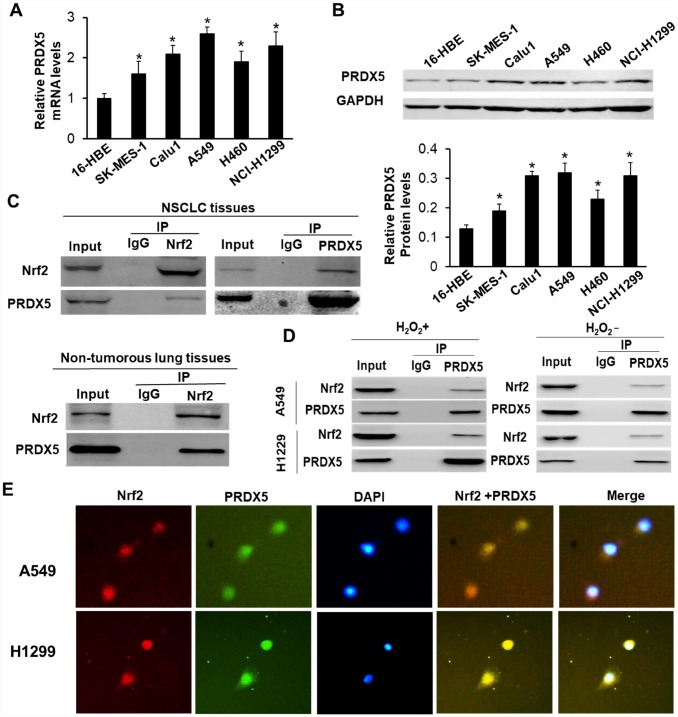
**PRDX5 interacted with Nrf2 in NSCLC tissues and related cell lines.** (**A**) qRT-PCR analysis of PRDX5 mRNA level in NSCLC cell lines and 16-HBE cells. The data are reported as the mean ± SD. *P < 0.05, compared with the level in 16-HBE cells. (**B**) PRDX5 proteins in the different NSCLC cell lines and the normal bronchial epithelial cell 16-HBE analyzed by Western blot analysis. The data shown represent the mean ± SD (*P < 0.05, compared with the level in 16-HBE cells). (**C**) Reciprocal immunoprecipitation of Nrf2 and PRDX5 in human NSCLC tissue (figure above) and PRDX5 was immunoprecipitated using an anti-Nrf2 antibody in the adjacent normal tissue (figure below). Lysates of the tissues were immunoprecipitated with anti-Nrf2, anti-PRDX5 antibodies or control IgG. The immunoprecipitates were subjected to Western blot analysis with anti-PRDX5 and anti-Nrf2 antibodies. (**D**) Interaction between Nrf2 and PRDX5 in A549 and NCI-H1299 cells under H_2_O_2_ treatment or nontreatment. The lysates obtained from the cells treated with 100 μM H_2_O_2_ for 30 min or not were immunoprecipitated using anti-Nrf2, anti-PRDX5 antibodies or control IgG. (**E**) Immunofluorescence analysis of Nrf2 and PRDX5 in A549 and NCI-H1299 cells. A549 and H1299 cells were pre-incubated with 100 μM H_2_O_2_ for 30 min, and then immunostained with a combination of anti-Nrf2 and anti-PRDX5 antibodies. The fluorescent images were digitally merged. Yellow coloration in overlay panels indicates colocalization of Nrf2 and PRDX5. Nuclei were counterstained with DAPI. Scale bar, 50 μm.

We first analyzed the expression of PRDX5 in both mRNA and protein levels in five lung cancer cell lines (A549, H460, NCI-H1299, Calu1 and SK-MES-1) by using qRT-PCR and Western blot method, respectively, and found that the expression of PRDX5 mRNA and protein were all higher than that in the normal bronchial epithelial cell 16-HBE, which increased the likelihood that PRDX5 may participated in the malignant progression of NCSLC (Figure 1A and 1B). Then, by immunoprecipitations, we found PRDX5 interacts with Nrf2 in both NSCLC tissues and cell lines. Figure 1C showed that PRDX5 can be brought down by an antibody of anti-Nrf2 from fresh NCSLC tissue lysate, and vice versa. At the same time, in PRDX5-expressing non-tumorous lung tissues, we also found that Nrf2 could be immune-precipitated by PRDX5, indicating that the binding of the two proteins was not limited to NCSLC tissues. When we treated A549 and H1299 cell lines with H_2_O_2_, we detected that there was obvious interaction between Nrf2 and PRDX5 (Figure 1D). Similarly, we also observed that the two proteins could bind to each other in untreated NCSLC cells. (Figure 1D). To further confirm Nrf2 and PRDX5 colocalization, immunofluorescence analysis was conducted in A549 and H1299 cells. The results showed Nrf2 and PRDX5 were partially co-localized in H_2_O_2_-treated NCSLC cells (Figure 1E). All these findings indicated that Nrf2 interacted with PRDX5 in NCSLC tissues and NCSLC cells.

### Nrf2-mediated recruitment of PRDX5 enhanced NQO1 expression

**Figure 2:**
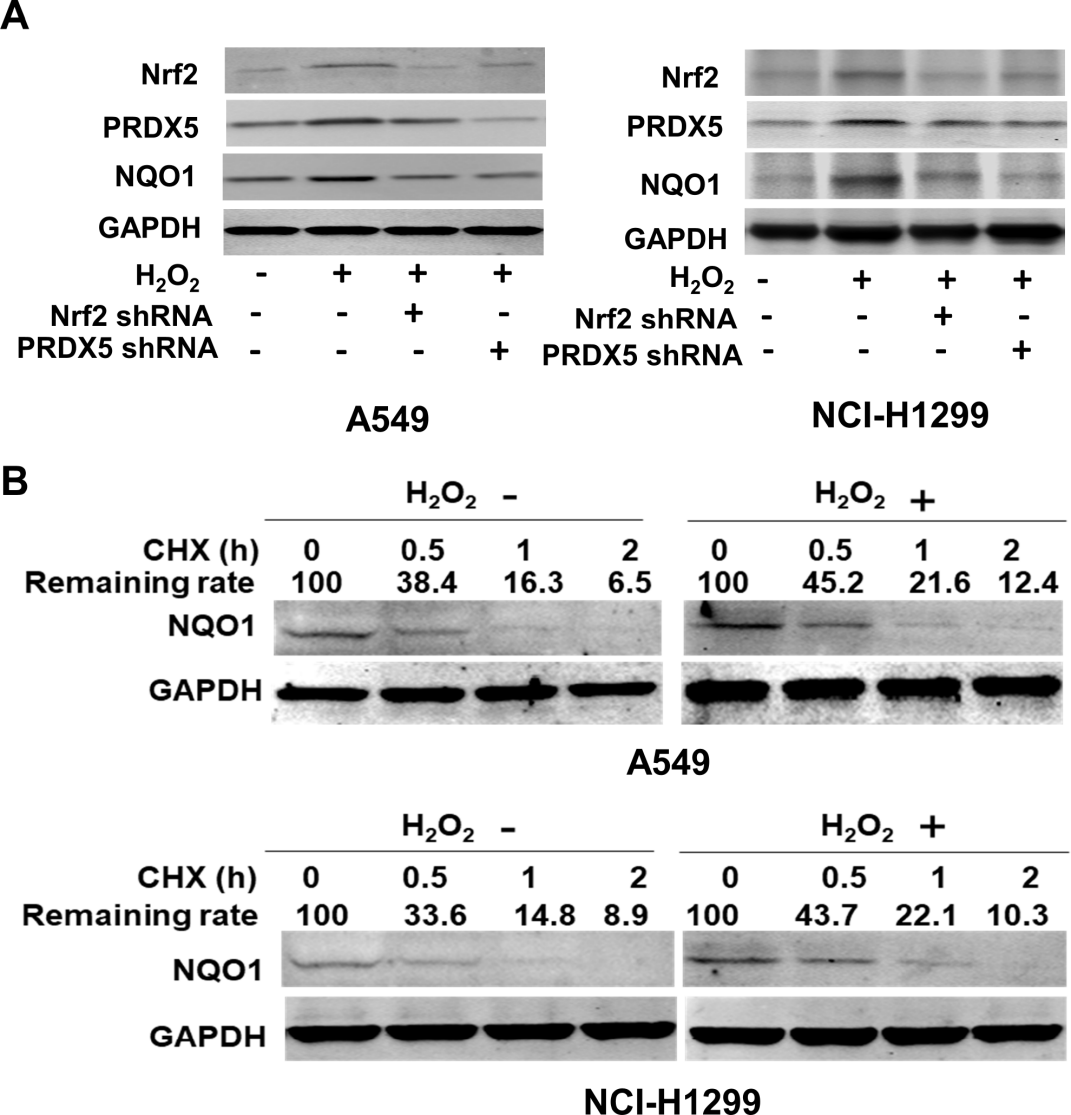
**The influence of Nrf2/PRDX5 on NQO1 expression.** (**A**) A549 and NCI-H1299 cells transfected into Nrf2 shRNA or PRDX5 shRNA were treated with serum-free medium overnight. The serum-starved cells were mock-treated, or stimulated with 100 μM H_2_O_2_ for 12 h. The expressions of Nrf2, PRDX5 and NQO1 were determined by Western blot. The data are mean ± SD (*P < 0.05). (**B**) After stimulated with 100 μM H_2_O_2_ for 12 h, A549 and H1299 cells were treated with 25 mg/L of cycloheximide (CHX) for the indicated period of time and subjected to Western blot analysis.

We treated A549 and H1299 cells with H_2_O_2_ and found the expression level of NQO1 protein increased significantly, while knockdown of Nrf2 reverse the upregulation of NQO1 protein in this circumstance of stimulation with H_2_O_2_ (Figure 2A). The above results showed that Nrf2 mediated the effect of H_2_O_2_. Similarly, PRDX5 knockdown significantly reduced NQO1 protein expression level in H_2_O_2_ treated A549 and H1299 cells (Figure 2A). Further, we try to use cycloheximide (CHX) chase experiment to clarify the mechanism underlying Nrf2/PRDX5-induced augmented NQO1 protein expression. The results showed that when treated with H_2_O_2_ or not in A549 and H1299 cells, the half-life time of NQO1 protein performed equally, and the results indicated that enhanced NQO1 protein expression stimulated with H_2_O_2_ did not occur at its post-translational level (Figure 2B). In sum, we clarified that Nrf2-mediated recruitment of PRDX5 enhanced NQO1 expression in NCSLC cells from the above results.

### Depletion of PRDX5 suppresses Nrf2-mediated cell proliferation

**Figure 3:**
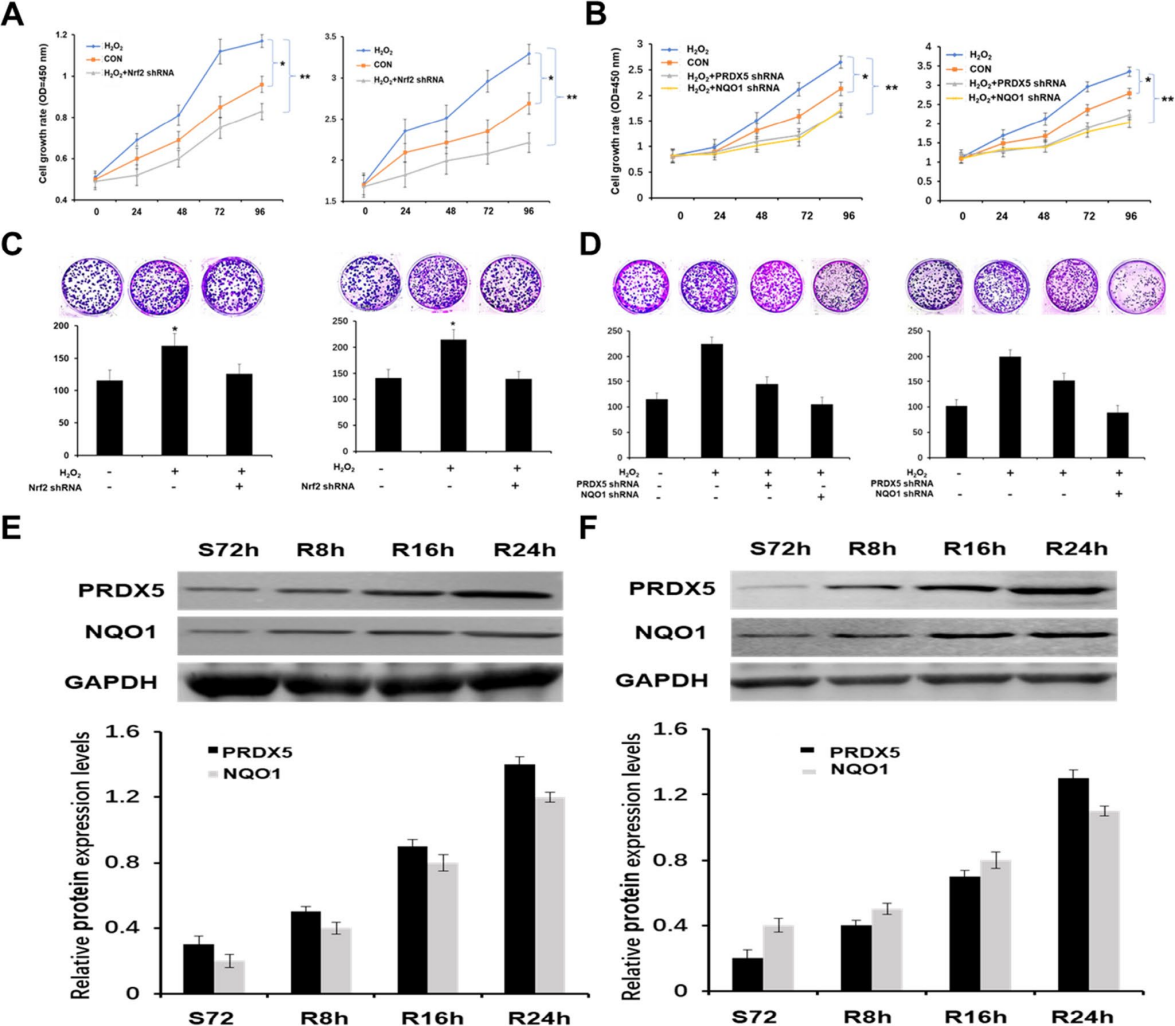
**Nrf2 enhanced the growth of NSCLC cells by PRDX5 and NQO1.** (**A**) Growth curves of A549 and H1299 cells treatment with 100 μM H_2_O_2_ or knockdown of Nrf2 using CCK-8 assay. All the data are mean ± SD and representative of three independent experiments (*P < 0.05). (**B**) Effect of PRDX5 or NQO1 knockdown on proliferation of A549 and H1299 cells in the presence of 100 μM H_2_O_2_ analyzed by CCK-8 assay. The data are reported as the mean ± SD of three independent experiments (*P < 0.05). (**C**) Nrf2 had an effect on the colony formation ability of NSCLC cells. Equal numbers of A549 and H1299 cells after treatment as above were seeded onto 6-well plates. The cells were fixed and stained with crystal violet after 14 days. The cell colonies (>0.5 mm in diameter) were counted after staining (mean ± SD, *P < 0.05). (**D**) Colony-forming capability was measured by colony formation assay in A549 and H1299 cells after transfected with PRDX5 shRNA or NQO1 shRNA and stimulated with 100 μM H_2_O_2_. The bar chart showed the number of colonies (>0.5 mm in diameter) in A549 and H1299 cells (mean ± SD, *P < 0.05). (**E** and **F**) A549 and H1299 cells were serum starved for 72 h, and then refed with serum for 0, 8, 16 and 24 h. The cell lysates of the corresponding time point were prepared and analyzed by Western blot using antibodies against PRDX5 and NQO1. GAPDH was used as a loading control. The bar charts demonstrated the ratio of PRDX5 or NQO1 to GAPDH by densitometry in A549 and H1299 cells. Mean ± SD of three independent experiments.

**Figure 4:**
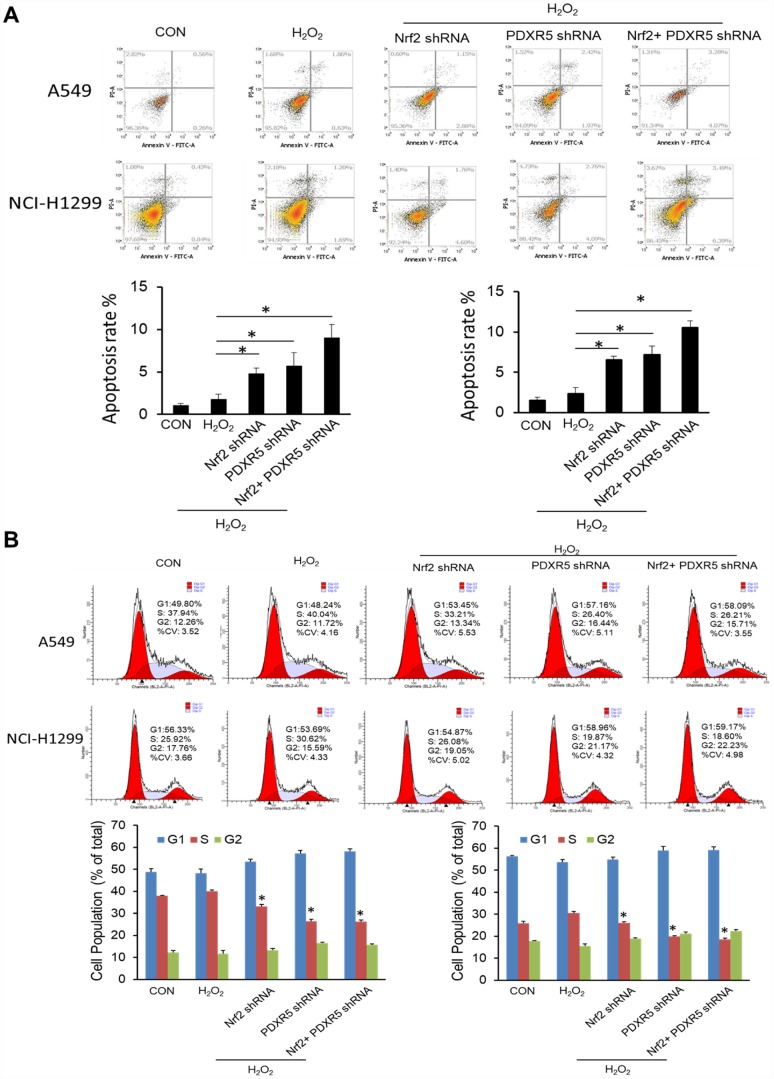
**The effects of Nrf2 and/or PRDX5 shRNA on cell proliferation by EdU incorporation assay.** (**A**) Nrf2 and/or PRDX5 shRNA significantly decreased cell proliferation ratio of A549 cells treated with H_2_O_2_ (*P < 0.05). (**B**) Nrf2 and/or PRDX5 shRNA significantly decreased cell proliferation of NCI-H1299 cells treated with H_2_O_2_ (*P < 0.05).

**Figure 5:**
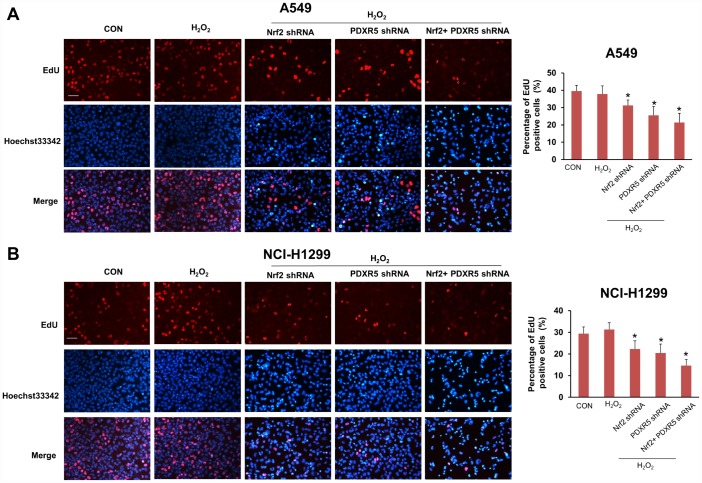
**The effects of Nrf2 and/or PRDX5 shRNA on cell proliferation and apoptosis of A549 and H1299 under oxidative stress by using flow cytometry.** (**A**) Nrf2 and/or PRDX5 shRNA significantly increased apoptosis ratio of A549 and H1299 cells treated with H_2_O_2_ (*P < 0.05). (**B**) Nrf2 and/or PRDX5 shRNA significantly decreased cell proliferation of A549 and H1299 cells treated with H_2_O_2_ (*P < 0.05).

We first tested and verified the impact of Nrf2 shRNA on the proliferation of NCSLC cells. The results of CCK-8 assay showed that the group treated with H_2_O_2_ elicited a significant increase in the proliferation of A549 and H1299 cells, while knockdown of Nrf2 with shRNA suppressed the proliferating effect obviously (Figure 3A). Colony formation assay also indicated the same effect of Nrf2 shRNA on cell proliferation *in vitro* (Figure 3B). Then we analyzed PRDX5 and NQO1 expression pattern in different proliferating statuses of NCSLC cells. The results showed that the protein levels of PRDX5 and NQO1 were increased gradually in released both A549 and H1299 cells after 72h of serum starvation (Figure 3C). These data support the conception that PRDX5 and NQO1 played important roles in regulating NCSLC proliferation. Next, we investigated whether the role of Nrf2 in promoting NCSLC growth is related to PRDX5 and NQO1. As shown in Figure 3D and 3E, the results illustrated that knockdown of PRDX5 or NQO1 significantly attenuated proliferation and colony formation capacity induced by H_2_O_2_ in A549 and H1299 cells, suggesting that Nrf2 may exert the effect of promoting proliferation through regulating PRDX5-dependent NQO1 expression. Then, we also analyzed the effects of Nrf2 and/or PRDX5 shRNA on cell proliferation and apoptosis of A549 and H1299 under oxidative stress by using flow cytometry. The results showed that Nrf2 and/or PRDX5 shRNA significantly increased apoptosis ratio of A549 and H1299 cells treated with H_2_O_2_ while decreased cell proliferation (Figure 4A, 4B). Subsequently, we further employed the EdU incorporation assay to determine the effects of Nrf2 and/or PRDX5 shRNA on cell proliferation. The results also showed that Nrf2 and/or PRDX5 shRNA groups significantly decreased cell proliferation ratio of A549 and NCI-H1299cells treated with H_2_O_2_ (Figure 5A, 5B).

### Clinical correlation of PRDX5 and Nrf2 expression in NCSLC tissues

**Figure 6:**
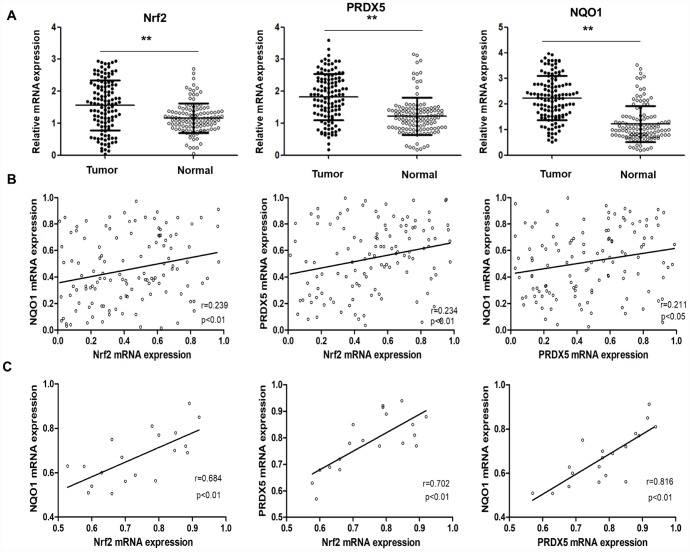
**The correlation of Nrf2, PRDX5 and NQO1 mRNA expression in NSCLC specimens.** (**A**) Nrf2, PRDX5 and NQO1 mRNA expressions in both tumor tissues and the adjacent normal tissues (**P < 0.01, compared with the adjacent normal tissues). (**B**) Statistical correlations among Nrf2, PRDX5 and NQO1 expressions in NSCLC samples. (**C**) Statistical correlations among Nrf2, PRDX5 and NQO1 expressions in NSCLC patients with Clinical TNM stage III-IV.

**Figure 7:**
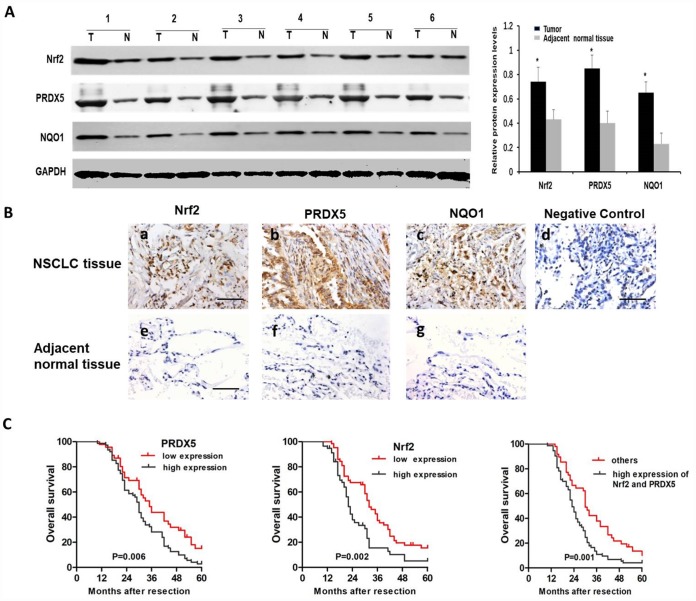
**The protein expressions of Nrf2, PRDX5 and NQO1 in NSCLC specimens.** (**A**) The Nrf2, PRDX5 and NQO1 expressions in 26 NSCLC and the adjacent normal tissues by western blot analysis. The representative western blot results in 6 cases are shown. GAPDH was used as a control for protein load and integrity. The bar chart demonstrated the ratio of Nrf2, PRDX5 and NQO1 expression to GAPDH between tumor and non-tumor tissues for the above by densitometry. *,^#^*P* < 0.05, significant upregulated expression of Nrf2, PRDX5 and NQO1 in cancerous tissues, compared with adjacent normal tissues. The data are reported as the mean ± SD (*, ^#^*P* < 0.05, compared with the adjacent tumor tissues). (**B**) Immunohistochemical analysis of Nrf2, PRDX5 and NQO1 in NSCLC tissues (**a**–**d**) and the adjacent normal tissues (**e**–**g**). Scale bar, 100 μm. (**C**) Cumulative survival curves according to Nrf2 and PRDX5 expression in 121 patients with NSCLC. Left, overall survival curves of low PRDX5 expression group vs high PRDX5 expression group (the choosing relative level of 2.323 as the optimal cut-off point of PRDX5). Middle, overall survival curves of low Nrf2 expression group vs high Nrf2 expression group (the choosing relative level of 2.825 as the optimal cut-off point of Nrf2). Right, overall survival curves of high Nrf2/PRDX5 expression group vs the other group.

To study clinical correlation, we first analyzed Nrf2, PRDX5 and NQO1 mRNA expression in both tumor tissues and the adjacent normal tissues, and the results showed upregulated these mRNAs in tumors (Figure 6A). The statistical correlations analysis among Nrf2, PRDX5 and NQO1 expressions in NSCLC samples showed that the level of PRDX5 expression in NCSLC tissues correlated with Nrf2 and NQO1 expression level very weakly (Figure 6B), while by subgroup of NSCLC patients with clinical TNM stage III-IV comparison, we found strong correlation among the three mRNA levels (Figure 6C). Further, we collected 26 pairs of matched fresh NCSLC and adjacent normal tissues to analyze PRDX5 and Nrf2 protein levels by using western blot. The results showed that the expression of PRDX5 increased significantly in cancer samples than that of in the non-tumorous samples, and the same trend of Nrf2 and NQO1 protein levels compared to the adjacent normal ones (Figure 7A). Moreover, we analyzed by immunohistochemistry analysis in 121 NCSLC specimens to further explore the clinical relevance between expression of Nrf2/PRDX5 and the clinicopathological factors (Figure 7B). The next study showed that PRDX5 and Nrf2 expressions were tightly associated with tumor size, clinical TNM stage, lymph node infiltration, differentiation and Ki-67 expression (Table 1).

**Table 1:** Correlation between Nrf2, PRDX5 expression and clinicopathological parameters in patients with NSCLC.

**Clinicopathological parameters**	**Total No.**	**Nrf2 expression**		**χ^2^**	**P Value**	**PRDX5 expression**		**χ^2^**	**P Value**
		**Low (%)**	**High (%)**			**Low (%)**	**High (%)**		
Age(years)				0.047	0.829			0.007	0.935
< 60	54	22(40.7)	32(59.3)			27(50.0)	27(50.0)		
≥ 60	67	26(38.8)	41(61.2)			34(50.7)	33(49.3)		
Gender				0.626	0.429			3.637	0.057
Male	58	19(32.8)	39(67.2)			24(41.4)	34(58.6)		
Female	63	25(39.7)	38(60.3)			37(58.7)	26(41.3)		
Tumor size(cm)				4.342	0.037*			11.569	0.001*
< 3	53	35(66.0)	18(34.0)			36(67.9)	17(32.1)		
≥ 3	68	32(47.1)	36(52.9)			25(36.8)	43(63.2)		
Smoking status				0.081	0.776			1.198	0.274
Yes	31	14(45.2)	17(54.8)			13(41.9)	18(58.1)		
No	90	38(42.2)	52(57.8)			48(53.3)	42(46.7)		
Histology				0.771	0.680			0.825	0.662
Adenocarcinoma	46	20(43.5)	26(56.5)			22(47.8)	24(52.2)		
Squamous cell carcinoma	53	24(45.3)	29(54.7)			26(49.1)	27(50.9)		
other	22	12(54.5)	10(45.5)			13(59.1)	9(40.9)		
Clinical TNM stage				4.221	0.040*			4.483	0.034*
I- II	102	53(52.0)	49(48.0)			57(55.9)	45(44.1)		
III- IV	19	5(26.3)	14(73.7)			5(26.3)	14(73.7)		
Lymph node infiltration				4.649	0.031*			5.307	0.021*
negative	73	45(61.6)	28(38.4)			43(58.9)	30(41.1)		
positive	48	20(41.7)	28(58.3)			18(37.5)	30(62.5)		
Differentiation				8.498	0.004*			11.807	0.001*
Well and moderate	103	53(51.5)	50(48.5)			60(58.2)	43(41.8)		
Poor and others	18	2(11.1)	16(88.2)			2(11.1)	16(88.2)		
Ki-67 expression				10.466	0.001*			12.052	0.001*
Low	74	25(33.8)	49(66.2)			28(37.8)	46(62.2)		
High	47	30(63.8)	17(36.2)			33(70.2)	14(29.8)		

Finally, Kaplan–Meier analysis showed that higher expressions of Nrf2 or PRDX5 were prone to obtain lower overall survival rates in NCSLC patients (*P* = 0.002 and 0.006), and high expressions of both Nrf2 and PRDX5 suggested poor prognosis of patients with NCSLC (*P* = 0.001, Figure 7C).

### Therapeutic effects of Nrf2 and PRDX5 shRNA on tumor growth *in vivo*

**Figure 8:**
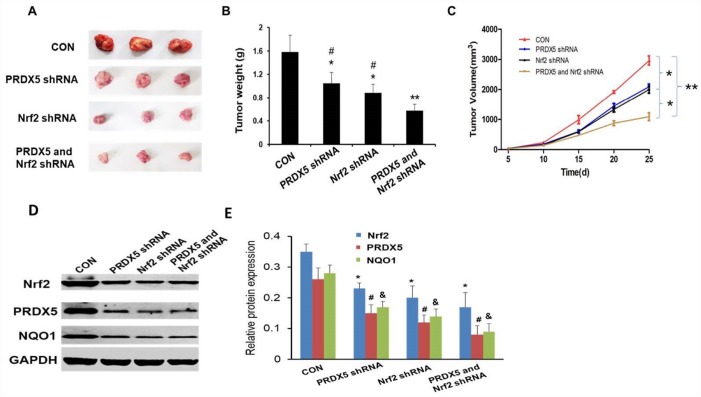
**Effect of Nrf2 and PRDX5 shRNA on tumor growth *in vivo*.** (**A**) Representative xenografts in each group. (**B**) The weights of xenograft tumors in mice after treatment with each shRNA. (*^,#^ *P* < 0.05, **P < 0.01). (**C**) Antitumor therapeutic efficacy in tumor bearing mice (*P < 0.05, **P < 0.01). (**D** and **E**) The protein expressions of Nrf2, PRDX5 and NQO1 in xenograft tumors in each group. (*^,#,&^ *P* < 0.05, compared with the control group, respectively).

We established human lung adenocarcinoma A549 xenografts mice models. In short, suspended A549 cells (5 × 10^6^) in 0.2 mL volume of Matrigel (BD Biosciences, San Jose, CA), then injected them into two flanks of nude mice subcutaneously. The nude mice were anesthetized when tumor volume gained to about 100 mm^3^, treated with Nrf2 and/or PRDX5 shRNA. After treatment period of 25 days cycle, the tumor volumes of Nrf2 and PRDX5 shRNA group were significantly decreased than that of the other groups and weights of the corresponding tumor were also the smallest (^*^P < 0.05, ^**^P < 0.01, Figure 8A–8C). Then we detected the protein expressions of Nrf2, PRDX5 and NQO1 in xenograft tumors in each group, and found the expression level of each protein in the treatment group was significantly lower than that in the corresponding control group (Figure 8D, 8E). The results indicated that Nrf2 and PRDX5 may be potential effective therapeutic targets.

## DISCUSSION

**Figure 9:**
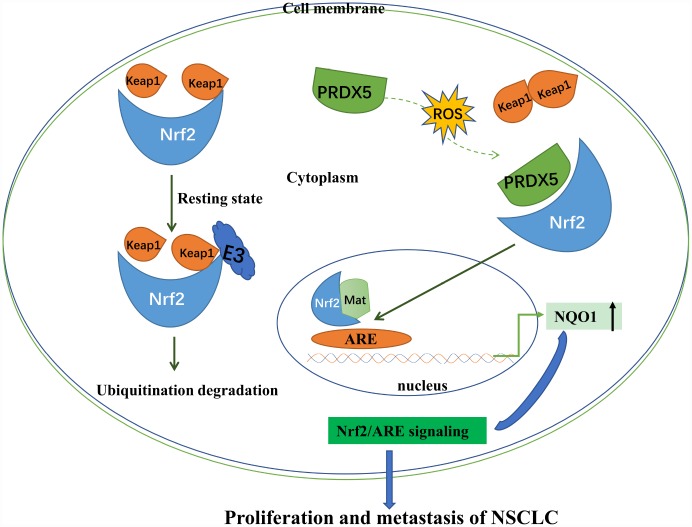
**Schematic showing that under condition of non-oxidative stress, Nrf2 binds to Keap1 and promotes ubiquitination of Nrf2.** Oxidative stress induces PRDX5 expression. Up-regulated PRDX5 competes with Keap1 for binding Nrf2 and inhibits ubiquitination of Nrf2 by Keap1. Up-regulation of Nrf2-ARE signaling pathway can promote proliferation and reduce apoptosis of NSCLC.

Changes in cytokines and transcriptional regulatory factors may alter the proliferation activity of cancer cells [23, 24], leading to differentiation or regulatory disorders of cancer cells, exacerbating the persistent progression of the lesion tissue in patients with lung cancer [25, 26]. In recent years, study of Nrf2-ARE signal pathway in NCSLC has been one of the hotspots. In the present study, we verified that under oxidative stress conditions, activated Nrf2 could recruit PRDX5 protein in NCSLC cells (Figure 9). Additionally, Nrf2-mediated recruitment of PRDX5 promoted the expression of NQO1 as well as NCSLC cell proliferation. Knockdown of PRDX5 and NQO1 significantly decreased H_2_O_2_-induced proliferation of NCSLC cells. Our results indicated that Nrf2 signaling may participate in the regulation of NCSLC growth by adjusting PRDX5-directed NQO1 expression.

Nrf2 plays an important role in influencing the activation of nuclear transcription promoters and cancer-associated signaling pathways in cancer cells, promoting cancer cell lesions, leading to the progression of lymph nodes or clinical stages of lung cancer lesions [27, 28]. In Nrf2 knockout mice, the absence of Nrf2 leads to chemically induced lung cancer more likely to occur, while in wild type mice, studies showed that the presence of Nrf2 may lead to a more significant proliferative effect in the already formed lung cancer cells that dependent on regulating the Kras pathway [29]. It is known that the level of Nrf2 in cells is mainly mediated by keap1-mediated ubiquitination. Once this regulation is broken, it can lead to an increase in Nrf2 expression, which involves all aspects of tumor proliferation, apoptosis, metastasis and drug resistance. However, it remains unclear whether transcription-independent mechanisms may contribute to Nrf2’s tumor-promoting role in NCSLC.

With interest in the biology of non-keap1 mutant NSCLC, we found a novel protein PRDX5, a protein that participates in translational regulation in the cytoplasm, can also interact with Nrf2, and showed enhanced interaction with Nrf2 under oxidative stress. The results implicated that PRDX5 could serve as a novel synergistic reaction molecule of Nrf2 to promote NCSLC development.

PRDX5 is an atypical two-Cys PRDX protein, and both the peroxidatic cysteine and resolving cysteine are critical for its catalytic activities [13]. Prdx5 regulates the amount of ROS produced in cells and protects them from ROS induced damage [30]. Recently, numerous studies have suggested that PRX5 was differentially expressed in various tumor tissues and enhanced tumorigenic phenotypes and metastatic potential [31–33]. In this study, we found that PRDX5 was upregulated in NCSLC cells and tissues, which implied that PRDX5 may exert an important role in regulating NCSLC growth. From experiment by NCSLC cell cultures, we found that knockdown of PRDX5 expression suppressed NCSLC cell proliferation as well as colony formation capacity.

NQO1 is a phase II detoxifying enzyme with anti-oxidative biological effects, which can catalyze the double electron reduction of quinone and inhibit the damage induced by oxidative stress reaction. It can also induce changes in tumor susceptibility after abnormalities. High NQO1 protein levels can be found in hepatocellular cancer [34], pancreatic ductal adenocarcinoma [35] and breast cancer [36], etc. In the present study, we verified that ROS increased protein level of NQO1 through pre-translational mechanisms by CHX chase experiment. Upregulation of NQO1 protein was induced by the release of Nrf2, while PRDX5 played a synergistic role in the effect through binding to Nrf2. Since the study showed high expression of PRDX5 and Nrf2 tightly associated with tumor size, clinical TNM stage, lymph node infiltration, etc, and predicted poor prognosis, we used Nrf2 and PRDX5 shRNA as therapeutic targets for tumor growth *in vivo* and achieved good results.

In sum, our studies demonstrated that PRDX5 can be a novel binding partner of Nrf2 in promoting NCSLC development under oxidative stress. Nrf2 upregulates NQO1 protein level by modulating PRDX5-mediated NQO1 expression. Further, activation of Nrf2 accelerates NCSLC cells proliferation through PRDX5/NQO1 signaling pathway. Moreover, high levels of Nrf2 and PRDX5 predict worsened outcome in patients with NCSLC, and Nrf2 and PRDX5 shRNA may be potential effective therapeutic targets. This study makes us further understanding of the molecular mechanisms by which Nrf2/PRDX5 signaling pathway regulates NCSLC pathogenesis. There are also some problems in this study that have not been resolved. Such as we are not sure if it is a direct interaction or not of PRDX5 and Nrf2 through *in vitro* experiment or does knock down PRDX5 cause ubiquitination degradation of Nrf2? We will focus on the follow-up studies.

## MATERIALS AND METHODS

### Patients and tissue specimens

NSCLC tissues and the adjacent normal tissues were collected from 121 patients who underwent lung cancer resection at Affiliated Hospital of Nantong University between 2009 and 2012. The Department of Pathology of Affiliated Hospital of Nantong University evaluated the tissue samples in accordance with World Health Organization standards. In addition, the clinical and follow-up data from patients’ medical records were collected. 26 pairs of fresh NSCLC and adjacent normal tissue samples were frozen in the liquid nitrogen immediately after surgical removal until using for Western blot analysis. The Ethics Committee of Affiliated Hospital of Nantong University (Jiangsu, China) approved the present study in accordance with the Declaration of Helsinki. Tissue samples from patients enrolled in the study were collected after obtaining informed consent signed by the patients or their family members.

### Cell lines

The human lung cancer cell lines A549, H460, NCI-H1299, Calu1, SK-MES-1 and the normal bronchial epithelial cell 16-HBE were purchased from the Shanghai Cell Bank of the Chinese Academy of Science (Shanghai, China). Cells were cultured in DMEM (Gibco, Grand Island, NY, USA) supplemented with 10% fetal bovine serum (FBS; Gibco), and incubated in a humidified atmosphere at 37°C and in a humidified atmosphere of 5% CO_2_.

### Nrf2 and PRDX5 shRNA

Nrf2 short shRNA lentiviral particles (sc-37030-V) and PRDX5 lentiviral particles (sc-40837-V) were purchased from Santa Cruz Biotechnology. The shRNA lentivirus was added to cultured cells for 24h, following by puromycin (5.0 mg/mL)-mediated selection for 10 days. In the stable cells over 95% Nrf2, PRDX5 or NQO1 knockdown was confirmed by Western blotting.

### Detection by qRT-PCR

The mRNA levels of PRDX5, Nrf2, NQO1 and GAPDH were analyzed by qRT-PCR using the SYBR Green Supermix kit (TaKaRa Bio, Shiga, Japan), GAPDH as internal reference, and the comparative cycle threshold (Ct) method was used to calculate relative amount of target mRNAs. Sequences of the primer for the genes are shown in Table 2.

**Table 2:** Primers used in qPCR.

**Gene**	**Primer sequence**
Nrf2-F	5′-ATGCCCTCACCTGCTACTTT - 3′
Nrf2-R	5′-AGGCCAAGTAGTGTGTCTCC - 3′
PRDX5-F	5′-CCAATCAAGACACACCTGCC - 3′
PRDX5-R	5′-TCTTGAGACGTCGATTCCCA - 3′
NQO1-F	5′-GGTGGAGTCGGACCTCTATG- 3′
NQO1-R	5′-ATATCACAAGGTCTGCGGCT - 3′
GAPDH-F	5′- CTGACTTCAACAGCGACACC - 3′
GAPDH-R	5′- GTGGTCCAGGGGTCTTACTC -3′

### Immunofluorescence assay

Immunofluorescence assay according the following steps: first the cells fixed in 4% paraformaldehyde for 0.5 hour at room temperature, PBS buffer washing for three times, and blocked with 5% donkey serum for 2 hours at room temperature, then incubated with anti-Nrf2 and anti-PRDX5 antibodies (Abcam) at 4°C overnight. PBS buffer washing for three times, the cells then were incubated with Alexa Fluor 488 or 594 conjugated secondary antibodies (Abcam) for 1 hour at room temperature. DAPI (Sigma-Aldrich) was used for nuclear staining. After adding anti-fluorescence quenching agent seal, the cells were visualized by the fluorescence microscopy (Leica DMR 3000; Leica Microsystem, Bensheim, Germany).

### Western blotting

The harvested cells or tissues were lysed in RIPA buffer containing protease inhibitors (Promega, Madison, WI). The concentration of total protein was determined by BCA protein assay (Bio-Rad, Hercules, CA). Then protein samples containing 30 μg of total protein were separated by SDS-PAGE gels and transferred onto polyvinylidene difluoride membranes. Membranes were blocked with 5% skimmed milk at room temperature for 1 hour and incubated overnight with the primary antibodies diluted 1:1000 at 4°C. Primary antibodies include PRDX5, Nrf2, NQO1, Flag, and GAPDH (all Abcam, USA). The next day, the membranes were further incubated with secondary antibodies (Rockland Gilbertsville, CA). The blots were scanned by Odyssey (Li-COR, USA) and taken the gray scale value for statistics.

### Coimmunoprecipitation

For coimmunoprecipitation, the supernatants of NSCLC tissue or cell lysates were incubated with the primary antibodies or control IgG in conjunction with Protein G Sepharose. Then the collected precipitates were separated and analyzed by Western blot.

### Immunohistochemistry

In brief, fix, embed, and cut tissue samples into sections about 4 μm thick. Then deparaffinize, hydrate, repair, and incubate them overnight at 4°C with anti-PRDX5, anti-Nrf2 and anti-NQO1 antibodies diluted in 1:50. Mayer's haematoxylin was used for nuclear counter staining. Two pathologists examined the immunostaining results independently, and the standard stage of tissue samples were divided as follows: 0 of no staining, 1 of weak, light yellow, 2 of moderate, yellow brown, and 3 of strong, brown. High expression as an intensity score of 2, and low expression as a score less than 2.

### Cell proliferation assay

For CCK8 detection, cells were cultured in 96-well plates (2 ×10^3^ cells per well) for 24 hours. The cell number was determined using the cell counting kit 8 (KeyGen, Nanjing, China) at 24, 48, 72, and 96 hours after shRNA transfection.

For 5-Ethynyl-2′-deoxyuridine (EdU) incorporation assay, in brief, cells of 4 × 10^3^ per well were cultured in 96-well plates for 48 h at 37 °C followed by 50 μM of EDU medium of 100 μL being added per well for another 2 hours at 37 °C, and then be fixed with 4% paraformaldehyde. The fixed cells were permeabilized with 0.5% Triton X-100 for 10 min followed by staining with Apollo® 567 and Hoechst33342, respectively.

### Clone formation assay

For plate clone formation assay, cells were plated in 6-well plates at a density of 1000 cells/dish with 5% CO_2_ at 37°C for two weeks. Then be washed with PBS, fixed for 15 min with paraformaldehyde and stained with 0.5% crystal violet. Finally, counted and analyzed the number of stained clones.

### Flow cytometry assay

The cells were trypsinized and harvested. Apoptosis was stained by using Annexin V-fluorescein isothiocyanate (FITC)/propidium iodide (PI) apoptosis kit (Invitrogen, Carlsbad, CA) and cell cycle was stained by a PI-based cell cycle kit (Invitrogen, Carlsbad, CA). Cells were assessed by Attune Acoustic Focusing Cytometer (Invitrogen).

### Animal study

The growth inhibited effect by PRDX5 and Nrf2 silencing on NCSLC cell line was determined *in vivo*. Nude mice were bought from the Experimental Animal Centre of Nantong University. The A549 cell was cultured, collected and washed with PBS buffer, then injected into the flanks of the mice subcutaneously (5×10^6^ cells/200 μL per side, 6 per group). The nude mice were anesthetized when tumor volume gained to about 100 mm^3^, injected with Nrf2 and/or PRDX5 shRNA. The tumor volumes were measured and calculated every 5 days using the following formula: volume V = width^2^ ×length/2. The experiments were approved by Nantong University Animal Care and Utilization Committee.

### Statistical analysis

Statistical analysis was performed by using SPSS 17.0 software package. All values were expressed as the mean ± standard deviation (SD). Differences were analyzed with one-way analysis of variance (ANOVA). When ANOVA detects significant differences, the data of above variables of each experimental group were compared with that of the control group using by a Dunnett’s t-test as post hoc test. The association between Nrf2 and PRDX5 and the clinicopathological factors was analyzed by using the Chi-square (χ^2^) test. Each experiment was repeated at least three times and differences were considered statistically significant at *P* < 0.05.
